# Supplementary material for: Dynamic architecture of mycobacterial outer membranes revealed by all-atom simulations
Source: eLife. 2026 May 6;14:RP108644. doi: 10.7554/eLife.108644 (PMC13148821; doi:10.7554/eLife.108644)
Supplement: Supplementary file 1. — System names indicate lipid composition and simulation temperature (K). Lipid composition is given as the number of molecules per leaflet in the order: MA_eU, MA_sZ, MA_W, PDIM, TDM, TMM, DAT, PAT, and SGL. Systems labeled ‘both’ are symmetric bilayers; asymmetric systems (Asym_313 and Asym_333) are reported with inner and outer leaflets listed separately. System size corresponds to the lateral box dimension (Å), membrane thickness to the bilayer thickness (Å), and APL to the area per lipid (Å²). Reported structural properties for asymmetric systems correspond to the full bilayer. [file elife-108644-supp1.docx]

**Supplementary File 1. System Compositions and Basic Statistics^†^.**

| **System Name (_Temp)** | **Leaflet** | **MA_eU : MA_sZ : MA_W : PDIM : TDM : TMM : DAT : PAT : SGL** | **System Size (Å)** | **Membrane Thickness (Å)** | **APL (Å^2^)** |
| --- | --- | --- | --- | --- | --- |
| MA_eU_313 | both | **60 : 00 : 00 : 00 : 00 : 00 : 00 : 00 : 00** | 62.4 | 59.4 | 64.9 |
| MA_eU_323 | both | **60 : 00 : 00 : 00 : 00 : 00 : 00 : 00 : 00** | 63.2 | 58.8 | 66.5 |
| MA_eU_333 | both | **60 : 00 : 00 : 00 : 00 : 00 : 00 : 00 : 00** | 63.6 | 59.0 | 67.5 |
| MA_eU_338 | both | **60 : 00 : 00 : 00 : 00 : 00 : 00 : 00 : 00** | 65.0 | 59.4 | 70.3 |
| MA_eU_343 | both | **60 : 00 : 00 : 00 : 00 : 00 : 00 : 00 : 00** | 66.0 | 59.2 | 72.6 |
| MA_eU_353 | both | **60 : 00 : 00 : 00 : 00 : 00 : 00 : 00 : 00** | 67.2 | 57.8 | 75.3 |
| MA_sZ_313 | both | **00 : 60 : 00 : 00 : 00 : 00 : 00 : 00 : 00** | 60.2 | 65.0 | 60.3 |
| MA_W_313 | both | **00 : 00 : 60 : 00 : 00 : 00 : 00 : 00 : 00** | 58.6 | 70.8 | 57.3 |
| MA_eU_W_313 | both | **30 : 00 : 30 : 00 : 00 : 00 : 00 : 00 : 00** | 61.9 | 61.7 | 63.9 |
| MA_sZ_333 | both | **00 : 60 : 00 : 00 : 00 : 00 : 00 : 00 : 00** | 64.4 | 61.2 | 69.2 |
| MA_W_333 | both | **00 : 00 : 60 : 00 : 00 : 00 : 00 : 00 : 00** | 64.4 | 61.4 | 69.0 |
| MA_eU_W_333 | both | **30 : 00 : 30 : 00 : 00 : 00 : 00 : 00 : 00** | 64.7 | 60.8 | 69.8 |
| All_Lipids_313 | both | **00 : 00 : 00 : 20 : 20 : 20 : 20 : 20 : 20** | 109.3 | 56.3 | 99.6 |
| No_PDIM_313 | both | **00 : 00 : 00 : 00 : 24 : 24 : 24 : 24 : 24** | 117.6 | 49.1 | 115.2 |
| No_TDM_313 | both | **00 : 00 : 00 : 24 : 00 : 24 : 24 : 24 : 24** | 106.0 | 53.8 | 93.6 |
| No_TMM_313 | both | **00 : 00 : 00 : 24 : 24 : 00 : 24 : 24 : 24** | 110.0 | 57.3 | 100.8 |
| No_DAT_313 | both | **00 : 00 : 00 : 24 : 24 : 24 : 00 : 24 : 24** | 113.7 | 57.4 | 107.7 |
| No_PAT_313 | both | **00 : 00 : 00 : 24 : 24 : 24 : 24 : 00 : 24** | 105.4 | 57.3 | 92.6 |
| No_SGL_313 | both | **00 : 00 : 00 : 24 : 24 : 24 : 24 : 24 : 00** | 102.8 | 60.1 | 88.0 |
| All_Lipids_333 | both | **00 : 00 : 00 : 20 : 20 : 20 : 20 : 20 : 20** | 106.3 | 59.6 | 94.1 |
| No_PDIM_333 | both | **00 : 00 : 00 : 00 : 24 : 24 : 24 : 24 : 24** | 113.0 | 54.5 | 106.3 |
| No_TDM_333 | both | **00 : 00 : 00 : 24 : 00 : 24 : 24 : 24 : 24** | 101.8 | 60.9 | 86.4 |
| No_TMM_333 | both | **00 : 00 : 00 : 24 : 24 : 00 : 24 : 24 : 24** | 106.7 | 62.0 | 94.8 |
| No_DAT_333 | both | **00 : 00 : 00 : 24 : 24 : 24 : 00 : 24 : 24** | 109.4 | 61.9 | 99.8 |
| No_PAT_333 | both | **00 : 00 : 00 : 24 : 24 : 24 : 24 : 00 : 24** | 105.5 | 56.5 | 92.8 |
| No_SGL_333 | both | **00 : 00 : 00 : 24 : 24 : 24 : 24 : 24 : 00** | 101.0 | 64.2 | 84.0 |
| Asym_313 | inner | **94 : 47 : 47 : 00 : 00 : 00 : 00 : 00 : 00** | 110.1 | 61.0 | 59.0 |
|  | outer | **00 : 00 : 00 : 20 : 20 : 20 : 20 : 20 : 20** |  |  | 92.4 |
| Asym_333 | inner | **94 : 47 : 47 : 00 : 00 : 00 : 00 : 00 : 00** | 105.3 | 63.8 | 64.5 |
|  | outer | **00 : 00 : 00 : 20 : 20 : 20 : 20 : 20 : 20** |  |  | 101.1 |

Legend: Composition and structural properties of simulated membrane systems. System names indicate lipid composition and simulation temperature (K). Lipid composition is given as the number of molecules per leaflet in the order: MA_eU, MA_sZ, MA_W, PDIM, TDM, TMM, DAT, PAT, and SGL. Systems labeled “both” are symmetric bilayers; asymmetric systems (Asym_313 and Asym_333) are reported with inner and outer leaflets listed separately. System size corresponds to the lateral box dimension (Å), membrane thickness to the bilayer thickness (Å), and APL to the area per lipid (Å²). Reported structural properties for asymmetric systems correspond to the full bilayer.

^†^The system size, membrane thickness, and area per lipid (APL) are the averaged numbers of the last 500 ns production.
